# Portrayals of Mental Health in Video Games: Protocol for a Scoping Review

**DOI:** 10.2196/78379

**Published:** 2026-04-29

**Authors:** Helga Dis Isfold Sigurdardottir, Víctor Manuel Perez-Colado, Cathrine Fredriksen Moe, Terje Geir Solvoll, Stefan Rennick-Egglestone

**Affiliations:** 1 Journalism and Creative Media, Faculty of Social Sciences, Nord University, Høgskolevegen 27, Levanger, 7600, Norway, 47 74 11 21 14; 2 Nord University, Levanger, Norway; 3 Faculty of Nursing and Health Sciences, Nord University, Bodø, Norway; 4 Norwegian Centre for E-health Research, Tromsø, Norway; 5 Institute of Mental Health, University of Nottingham, Nottingham, United Kingdom

**Keywords:** mental health challenges, video games, mental health portrayal, recovery narratives, leisure gaming

## Abstract

**Background:**

Digital games are increasingly influential in shaping public perceptions of mental health due to their interactive and narrative nature. While serious games designed for therapeutic use have been studied, less is known about how mental health challenges are portrayed in mainstream video games for leisure and the impact of these portrayals on players.

**Objective:**

This scoping review aims to explore how academic studies address the representation of mental health challenges in contemporary video games for leisure, with particular attention to game design features, depictions of coping or living with a mental health condition, recovery, and intervention.

**Methods:**

The review follows the PRISMA-ScR (Preferred Reporting Items for Systematic Reviews and Meta-Analyses Extension for Scoping Reviews) methodology and applies the population, concept, and context framework. A comprehensive literature search was conducted across 6 databases (PubMed, Scopus, ProQuest, ERIC, IEEE Xplore, and Web of Science) in July 2024 and will be updated to include studies published until November 2025. Eligible studies include peer-reviewed original research on the portrayal of mental health in mainstream digital games. Screening and data extraction were conducted independently by 2 reviewers using Rayyan and NVivo software. Data synthesis will be narrative, focusing on themes of representation, game mechanics, recovery, and audience impact.

**Results:**

As this is a protocol, no empirical results are available. The completed review will map how scholarly studies conceptualize and analyze portrayals of mental health in video games, identify recurring thematic and design patterns, and synthesize trends in how researchers frame the relationship between gameplay and mental health representation.

**Conclusions:**

By systematically mapping the current literature, this review will contribute to a deeper understanding of how mental health is depicted in mainstream games and will highlight areas for future research, including ethical considerations and opportunities for more informed, empathetic, and responsible game design.

## Introduction

### Rationale

The portrayal of mental health challenges in video games is an important area of inquiry in media and mental health studies due to the immersive nature of digital gameplay [1,2]. This review aims to critically examine how academic research has studied the portrayal of mental health challenges in mainstream, leisure-oriented digital games.

Although the use of games for serious purposes is not new, video games that are developed with the specific aim of being used for educational, therapeutic, or rehabilitation purposes are often referred to as serious games [1,3]. Unlike serious games with explicit educational or therapeutic intent, mainstream games reach broader audiences and often influence perceptions more subtly [2]. Understanding these portrayals is vital for recognizing the societal messages embedded in entertainment media. However, research on mental health representation in games remains limited, and existing frameworks, largely adapted from film and television studies, often fail to account for the unique interactive and design-based dimensions of digital play [4]. This dimensional perspective underscores the need for more systematic investigation of how games construct and communicate experiences of mental illness.

Recent media analyses underscore that representation shapes public perceptions of health and illness. For instance, a recent (2025) quantitative analysis demonstrated that cinematic portrayals of mortality diverge sharply from real-world data [5]. This reinforces the broader point that what media choose to depict or omit can distort societal understandings of health and identity. Similarly, a systematic review from 2025 showed that how games depict and frame mental health concepts can influence players’ understanding and engagement with these issues [6]. In contrast, an exploratory study, also from 2025, found no significant relationship between playing commercial games depicting mental illness and holding stigmatizing beliefs [7]. However, the exploratory study also highlights how limited the existing evidence base remains, as most research to date examines individual titles or narrow subsets of games [7]. Therefore, a comprehensive synthesis of the broader scholarly literature is needed to understand how portrayals of mental health in games have been studied across contexts and disciplines.

In this review, we use the term “mental health challenges” to encompass a broad range of experiences that impact psychological well-being. This includes both clinically recognized mental illnesses (eg, schizophrenia and depression) and nonclinical forms of emotional distress (eg, grief and emotional crisis). This inclusive approach is aligned with recovery-oriented research [8], the World Health Organization’s continuum model of mental health [9], and the NEON Young Norway project’s focus on diverse recovery narratives [10].

The review synthesizes how academic studies describe the narrative and design mechanisms that shape portrayals of mental health in games, as well as how researchers link these portrayals to empathy, stigma reduction, and cultural discourse.

### Objectives

This scoping review is guided by 1 overarching research question, supported by 3 follow-up questions designed to explore specific aspects of the topic in greater depth.

### Main Research Question

The main research questions are as follows: How have academic studies portrayed and analyzed the representation of mental health challenges in contemporary video games for leisure, andwhat roles do game mechanics and design features play, according to this literature?

### Follow-Up Questions

The follow-up questions are as follows:

- How do existing studies describe the portrayal of interventions related to mental health– challenges in these games?
- How do researchers conceptualize recovery, acceptance, and coping mechanisms in game-based portrayals of mental health?
- How do studies assess the perceived impact of these portrayals on public perception, including their potential to influence stigma or mental health literacy?

These questions align with the scoping review’s aim to map the range, nature, and complexity of mental health representations in mainstream digital games as studied in the academic literature and to identify the interpretive frameworks through which these portrayals are analyzed and discussed. The review does not focus on specific participant groups (eg, age or diagnosis) but on how researchers have examined representations within games themselves.

## Methods

### Recruitment

Recruitment criteria are not applicable, as the focus is on published research on games rather than human participants.

### Eligibility Criteria

#### Population, Concept, and Context Framework

The eligibility criteria were defined using the population, concept, and context framework. The population is all populations represented in or addressed by mainstream digital games. The review does not focus on primary human participants but rather on the games themselves as artifacts that embed, reflect, or target specific player populations. This inclusive definition allows for mapping the full breadth of existing peer-reviewed literature on the topic. The concept is portrayals of mental health challenges (this is broadly defined and includes “mental,” “psychological,” “psychiatric,” “emotional,” and derivatives). The context is mainstream, leisure-oriented digital games (including video, computer, electronic, and social games)

#### Types of Sources

The primary source type considered for this study is peer-reviewed original research (qualitative, quantitative, and mixed methods). Literature reviews, protocols, gray literature, and non-English publications are excluded. Studies that primarily examine psychological or behavioral effects of gameplay (eg, addiction, aggression, or depression among players) are excluded, as the focus is on how mental health challenges are portrayed within games themselves rather than on their effects on players.

### Information Sources

Initial searches were conducted in PubMed, Scopus, ProQuest, ERIC, IEEE Xplore, and Web of Science in July 2024. Following protocol peer review, an updated search will be conducted prior to final synthesis to include studies published up to November 2025. No restrictions were placed on publication year due to the emerging nature of the topic.

### Search Strategy

An initial exploratory search helped define key terms, and the full search strategy was developed in collaboration with a university librarian. The searches used keywords and index terms related to mental health, representation, and digital games.

Terms related to therapy and rehabilitation were excluded to maintain a focus on leisure-oriented contexts, whereas “serious games” was retained due to inconsistent usage of this term across the literature, sometimes referring to mainstream titles that address serious topics rather than explicitly therapeutic or rehabilitative games. However, studies focusing on games designed primarily for therapeutic or rehabilitative purposes were excluded, as the review targets mainstream leisure-oriented games with potential societal and cultural influence through entertainment contexts. Accordingly, exclusion terms such as “therap*,” “rehabilit*,” “engineering,” and “computational” were applied to filter out intervention-focused and technical design studies beyond the review’s scope. The final query retrieved studies that included a direct reference to video games (and 6 related synonyms) in the title, abstract, keywords, or subject fields and a direct mention of mental health and representation anywhere in the text. In the exclusion component of the query, we filtered out papers referring to therapy, rehabilitation, or (game) addiction, as this review focuses on games designed for leisure. Literature reviews were excluded; however, reviews of games themselves (ie, studies systematically analyzing game content rather than academic publications) were eligible for inclusion. Papers emphasizing the engineering or computational aspects of game development were excluded. The complete “mother query,” including all synonyms and exclusions, is shown in Textbox 1.

Textbox 1. **Mother query, including all synonyms and exclusions.**

**Title/abstract/keywords/themes**

(“Video gam” OR “Computer gam” OR “Digital gam” OR “Electronic gam” OR “Serious gam” OR “Social impact gam”) AND (Mental OR Psych* OR Emotion* OR Neuro*) AND (portray* OR depict* OR narrat* OR adventur*) AND subject (“Video gam” OR “Computer gam” OR “Digital gam” OR “Electronic gam” OR “Serious gam” OR “Social impact gam”)

**Full text**

NOT (therap* OR rehabilit* OR addictive OR treatment OR “mental calculation” OR “mental model” OR “Literature review” OR “Meta-Analysis” OR “quantitative review” OR “Systematic Review” OR “Scoping Review” OR “Narrative Review” OR “Critical Review” OR “Realist review” OR “Literature mapping” OR “Integrative Review” OR “Umbrella Review” OR “State-of-the-Art Review” OR “Qualitative Review” OR “Rapid Review” OR “Mapping Review” OR “Evidence Synthesis” OR “book review” OR engineering OR computational OR technical OR “deep learning” OR market OR politic* OR “game* addict” OR “gam disorder” OR “physical training” OR “Independent component analysis” OR “pattern recognition” OR “natural science”)

### Study Records

#### Data Management

Records were managed in EndNote (version 20; Clarivate) [11]. Duplicates were removed, and the remaining references were uploaded to Rayyan (Rayyan Systems Inc) for screening [12].

#### Selection Process

Two reviewers (HDIS and VMP-C) independently screened titles and abstracts, followed by full-text screening. Discrepancies were resolved through discussion with a third reviewer (CFM).

#### Data Charting Process

A structured data charting process was developed and implemented using NVivo (version 15; Lumivero). Each included study was imported as a source and assigned a corresponding case with a standardized set of attributes (eg, authorship, publication year, study type, games analyzed, population, country, and focus on recovery or interventions; refer to the Data Items section for full list). This structured metadata allowed for easy filtering and comparison across studies.

A hierarchical coding framework was developed based on the review’s research questions, including categories for mental health conditions portrayed, narrative and design mechanisms, depictions of recovery, coping mechanisms, therapy and impact, ethical considerations, and audience impact. Textual segments were coded to relevant nodes, and memos were used to document coding decisions, emergent insights, and reviewer reflections.

The charting framework was piloted on a subset of studies and refined through team discussion. HDIS and VMP-C independently coded the studies, with regular meetings to resolve discrepancies and maintain consistency. The structured use of NVivo enables both qualitative depth and systematic comparison, supporting the narrative synthesis of findings across thematic domains.

### Data Items

Extracted items include authors, year, country, aim, journal, study design, methods, games analyzed, types of mental health representations, narrative themes, game mechanisms, ethical considerations, and theoretical frameworks used for analysis, where relevant.

### Outcomes and Prioritization

The primary and secondary outcomes are summarized in Textbox 2.

Textbox 2. **Primary and secondary outcomes and prioritization.**

**Primary outcomes**

- Narrative and design mechanisms used to portray mental states and mental health challenges
- Themes and patterns in the representation of mental health challenges in video games
- Thematic representation of recovery and coping
- Representation of therapy and other interventions
- Impact on players and public perception

**Secondary outcomes**

- Ethical considerations in representation
- Trends in how researchers conceptualize mental health challenges in games (eg, diagnostic categories and metaphorical portrayals)

### Data Synthesis

Quantitative synthesis is not planned. A narrative synthesis will be provided, structured around key themes and concepts identified during data charting. Tables will be used to summarize the included studies.

### Meta-Biases

Meta-bias assessment is not applicable. However, we will consider publication and disciplinary bias during the interpretation of the results.

### Confidence in Cumulative Evidence

As this is a scoping review, we do not intend to formally assess the quality of evidence using tools such as GRADE (Grading of Recommendations Assessment, Development, and Evaluation), which are designed for evaluating intervention studies. Instead, we aim to map the scope, nature, and thematic diversity of the existing literature. We will not assess the accuracy, clinical validity, or ethical adequacy of the portrayals of mental illness in games. Rather, we will describe how mental health challenges are represented, which topics are most frequently addressed, and how researchers have approached these representations across various disciplinary perspectives.

### Ethical Considerations

This review involves the analysis of previously published literature and does not include human participants or primary data collection. Therefore, ethics approval was not required, in accordance with institutional and national guidelines.

### Target Journal

*JMIR Mental Health,* or a comparable open-access journal focusing on digital health and media research, is the target journal. Submission is planned following final team approval and registry completion.

## Results

**Figure 1.**
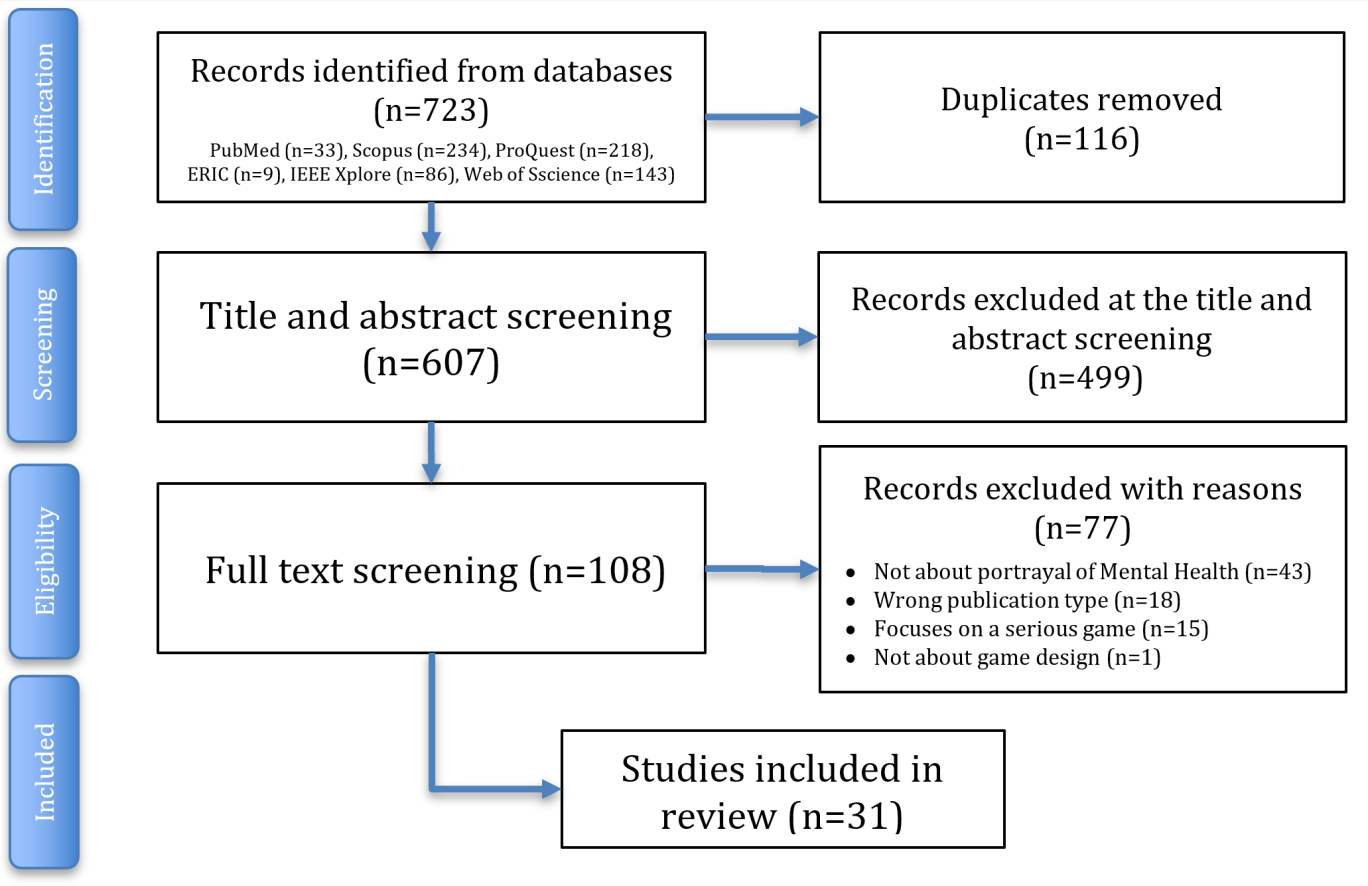
PRISMA (Preferred Reporting Items for Systematic Review and Meta-Analysis) flow diagram illustrating the identification, screening, and inclusion process for the initial literature search (July 2024). An updated search is planned for November 2025 to capture any literature published during the review process.

Initial database searches were completed in July 2024 across 6 databases (PubMed, Scopus, ProQuest, ERIC, IEEE Xplore, and Web of Science). Following deduplication, titles and abstracts were screened in Rayyan by 2 independent reviewers. The study selection and screening process is illustrated in Figure 1.

In the first screening round, 31 studies were initially identified as eligible and analyzed in NVivo to pilot and refine the coding and data charting framework; 27 of these were ultimately included in the analysis. At the time of protocol revision, coding of these records had been completed by 1 reviewer, while analysis by the second reviewer was underway. An updated search will be conducted prior to final synthesis to extend the inclusion period through November 2025.

An updated search will be planned before finalizing the full scoping review to extend the inclusion period through November 2025, in response to reviewer recommendations to incorporate the most recent literature. This update is not expected to substantially alter the scope or direction of the review.

Completion of dual coding, synthesis, and manuscript preparation is anticipated by early 2026. This manuscript represents a revised iteration of the protocol following peer review feedback. Initial database searches, screening, and preliminary coding of included studies in NVivo have been completed, and the review process is ongoing. The protocol continues to serve as a transparent methodological record for the remaining stages of analysis and synthesis. By documenting refinements made in response to reviewer input, this protocol supports transparency, reproducibility, and critical appraisal.

## Discussion

### Principal Aims and Anticipated Contribution

This scoping review will map how scholarly studies describe portrayals of mental health challenges in leisure-oriented video games and how narrative, aesthetic, and mechanical design choices shape those portrayals and their reception. We hypothesize that the literature may predominantly report stereotypical or negative depictions (eg, violence, danger, and institutionalization), with comparatively fewer “dimensional” portrayals that integrate mental health experiences across story, mechanics, and art direction. We also expect relatively sparse and unfavorable depictions of formal interventions, alongside a small set of cases—frequently from independent titles—that are highlighted for empathy, acceptance, or constructive coping. The review will synthesize thematic patterns and the interpretive frameworks used by researchers, and it will summarize reported impacts on public perception (eg, empathy, stigma, and mental health literacy).

### Positioning in Relation to Prior Work

This review is intended to complement and extend existing overviews of mental illness in commercial games by moving beyond counts or typologies of diagnoses to examine how portrayals are produced through design (eg, player perspective, agency constraints, procedural rhetoric, and audiovisual cues) and how studies interpret audience effects [1,2,7]. Prior work has documented how in-game portrayals can influence players [3,5,6]; however, it also indicates a predominance of negative and stereotypical depictions [1,2]. Recent research has further emphasized that studies on mental health representation in games remain limited and often lack frameworks that account for medium-specific design features [4]. This review will also incorporate recovery- and intervention-oriented perspectives that are underrepresented in earlier syntheses. By updating the search window to late 2025, the review aims to capture newer analyses and to compare convergences and divergences with prior mappings of the field, including areas in which independent titles have been argued to provide more nuanced representations than mainstream releases.

### Implications and Future Directions

A structured map of representational patterns and design mechanisms can inform both scholarship and practice. For researchers, the synthesis may help identify gaps suitable for targeted empirical work (eg, experiments on stigma reduction and narrative transportation, longitudinal studies of attitude change, or cross-cultural comparisons of reception). For game developers and educators, the review can surface design patterns associated with more humanizing portrayals (eg, player identification, scaffolded agency, and reflective mechanics) and common pitfalls (eg, pathologizing archetypes and punitive institutional tropes). Findings may be relevant to public mental health communication, suggesting opportunities for co-design with people with lived experience and for integrating recovery-oriented narratives into entertainment media. Because the review focuses on published research rather than direct game analysis, it necessarily excludes popular titles that have not yet been examined in academic literature. This gap represents an opportunity for future research.

### Limitations and Dissemination

As a scoping review, this study will not appraise study quality or make claims about efficacy; it will describe the breadth and main characteristics of scholarship on portrayals of mental health in games. The corpus is limited to peer-reviewed, English-language publications and therefore may underrepresent non-English and gray literature perspectives. Gray literature was excluded to ensure consistency in study quality and verifiability. Although this decision may omit practitioner or popular perspectives, it allows for a focused synthesis of peer-reviewed research and facilitates methodological transparency and reproducibility. Because the unit of analysis is research about games rather than the games themselves, the sample necessarily includes only titles that have attracted academic analysis. Publication and disciplinary biases will be considered when interpreting patterns. We plan to disseminate the protocol and subsequent review through JMIR Publications or comparable open-access journals, conference presentations, and a publicly shareable codebook (and, where feasible, aggregated charting outputs) to support transparency and reuse. The planned search update through November 2025 will ensure inclusion of the most recent studies prior to synthesis.

## Abbreviations

GRADE: Grading of Recommendations Assessment, Development, and Evaluation
